# Sexual stature difference fluctuations in pre‐ and post‐Black Death London as an indicator of living standards

**DOI:** 10.1002/ajhb.23783

**Published:** 2022-07-19

**Authors:** Emily J. Brennan, Sharon N. DeWitte

**Affiliations:** ^1^ Department of Anthropology University of South Carolina Columbia South Carolina USA

## Abstract

**Objectives:**

The degree of sexual stature difference (SSD), the ratio of male to female height, is argued to be an indicator of living standards based on evidence that physical growth for males is more sensitive to environmental fluctuations. In a resource‐poor environment, the degree of SSD is expected to be relatively low. The aim of this study is to comparatively assess SSD in medieval London in the context of repeated famine events and other environmental stressors before the Black Death (BD) and the improved living conditions that characterized the post‐Black Death period.

**Methods:**

To test the hypothesis that a poor nutritional environment resulted in decreased SSD in medieval London, this study compares adult individuals from early pre‐Black Death (*c.* 1000–1200), late pre‐Black Death (*c.* 1200–1250) and post‐Black Death (*c.* 1350–1540) cemetery contexts from London. Maximum tibial,femoral, and lower limb lengths were used as a proxy for stature, and SSD was calculated using the Chakraborty and Majumber index.

**Results:**

Compared to the late pre‐BD period, we find a slighter higher degree of SSD in the post‐BD period for all three stature proxies used. This increase is attributed to more exaggerated increases in stature for estimated males post‐BD.

**Conclusions:**

This study demonstrates the importance of examining variables that are considered indicators of living standards in light of factors like selective mortality, catch‐up growth, and urban migration patterns. Future research needs to further investigate how cultural and biological processes influence the mechanisms that produce adult stature.

## 1 INTRODUCTION

Due to the links between growth, health, and mortality, adult stature has long been used by researchers as a marker of well‐being and health in past and present populations (Gunnell et al., 2001; Kemkes‐Grottenthaler, 2005; Perkins et al., 2016; Silventoinen, 2003; Silventoinen et al., 1999). While adult height is heritable to a certain extent (Livshits et al., 2002), environmental factors, especially those affecting nutrition, can constrain an individual's ability to reach their full height potential. Exposure to stress during development, such as malnutrition or infection, can influence achieved adult stature (Haviland, 1967; Powell, 1988; Steckel, 1995). In humans, undernutrition in the fetal and immediate post‐fetal environment can change growth trajectories (Bateson et al., 2004) via processes of developmental plasticity. Developmental plasticity represents an organism's ability to alter its phenotype in response to environmental conditions (Bateson et al., 2014; Gluckman et al., 2009), and this adaptive response can either trigger or delay developmental pathways during critical periods of growth (Bateson et al., 2004). Developmental plasticity processes are hypothesized to influence achieved adult stature through “brain sparing” mechanisms that redirect resources toward vital organs and away from limbs during resource scarce periods of development (Barbiro‐Michaely et al., 2007; Giussani, 2011).

Relatively short adult stature is positively associated with risk of infectious disease, possibly, at least in part, because chronic stress during critical periods also influences immune response to infection and disease later in life (Barker, 1990; Barker, 2006, p. 200; Gluckman et al., 2007; Moore et al., 1999; Perkins et al., 2016). In a metadata analysis, Paajanen et al. (2010) concluded that a link between short stature and increased risk of cardiovascular morbidity and mortality exists for men and women; adults within the shorter stature category had a 50% higher risk of coronary heart disease morbidity and mortality compared to tall individuals. Given this connection, understanding developmental plasticity pathways and the biological and cultural influences that impact adult height in conjunction with health can help in the crafting of effective public health interventions (Schroeder et al., 2002).

Both adult stature and the degree of sexual stature differences have been utilized to investigate health and well‐being in both living and past populations. The term “well‐being,” as utilized in bioarchaeological research, includes variables such as life expectancy, nutritional status, and health at both the individual and population level (Wood, 1998). Here, we use height differentials as an indicator of stress and a measurable element of health. In humans, sexual stature bimodalism or difference is defined as the difference in average male stature relative to average female stature. Many factors are thought to influence the degree of SSD in a population including diet, sexual division of labor, mating systems, and climate (Holden & Mace, 1999; Plavcan, 2001; Pomeroy & Zakrzewski, 2009; Shine, 1989). Ultimately, these factors affect the achieved adult height of females and males by influencing environmental conditions present during growth and development. Therefore, the use of sexual stature difference specifically as an indicator of living standards is grounded in sex differences in stature that are presumed to occur in response to environmental factors. Females are hypothesized to be better equipped to buffer against environmental insults via inherent biological mechanisms (e.g., an enhanced immune response), whereas physical growth for males may fluctuate more with variation in environmental conditions (Grossman, 1989; Sohn, 2016; Stinson, 1985). Therefore, males who experience relatively good nutritional and environmental conditions are expected to be taller than males in poorer environmental conditions. In contrast, female height is not anticipated to fluctuate as dramatically with environmental conditions given the aforementioned buffering. As such, a higher degree of SSD in a population is therefore expected when environmental conditions are relatively good.

SSD has been investigated as a marker of “well‐being” in studies of both living and past populations with mixed results. For samples from early modern Estonia, the degree of estimated SSD was greater in rural populations compared to contemporary urban populations, suggesting better access to resources in the rural areas, especially during critical developmental periods (Allmäe & Limbo, 2010). Charisi et al. (2016) also found greater SSD for a rural sample compared to a contemporaneous urban sample in Medieval Spain, suggesting that females in the city were taller as a result of a more active role in working life that translated to better access to food and superior living conditions compared to rural females who were shorter on average. However, Gustafsson et al. (2007) found no change in SSD from the 10th to 17th century in Sweden, despite an increase in average adult height through the end of the 20th century, likely due to improved living conditions.

Since socioeconomic status can influence resource availability and therefore affect living conditions across the lifespan, the degree of SSD may be expected to fluctuate between class groups. A cohort study from 20th Spain using self‐reported height and socioeconomic information (using educational attainment as a proxy for status), found that middle‐upper classes exhibited higher values of SSD that are similar to the modern population earlier than did the lower SES group (Cámara, 2015). Similarly, low SSD in the Badarin population of predynastic Egypt (ca. 5000–3900 BC) and a higher degree in the Late Predynastic population (ca. 3500–3100 BC) is thought to follow patterns of social complexity, as the Badarin were more egalitarian while social hierarchy and ranking developed in the later period (Zakrzewski, 2003). However, Sohn (2016) found no increase in SSD for a representative sample of South Korean men and women, even when stratified by socioeconomic status, from 1941 to 1990 despite improvements in GDP per capita and life expectancy at birth.

Medieval London provides an opportunity to test the effect of overall environment on stature and degree of SSD. The 14th‐century Black Death was chosen as a demarcation for different environmental conditions in London based on both bioarchaeological and historical evidence of the nature of the socioeconomic and nutritional environments before and after the epidemic. The outbreak of bubonic plague that came to be known as the Black Death arrived in London in 1348 and within 18 months killed almost half the population of the city (Hawkins, 1990). Before 1348, multiple episodes of famine in the decades prior to the arrival of the Black Death and increasing social inequalities likely had negative effects on general population health. Overall cooling temperatures experienced in Europe during the 13th and 14th centuries contributed to widespread famine events in England like the Great Famine of 1315‐1317 and the Great Bovine Pestilence of 1319‐1332, which compromised crop production and milk outputs (Büntgen et al., 2011; Slavin, 2012). Prior research has indicated that the Black Death was not an indiscriminate killer, rather than older adults and individuals of all ages who were frailer (i.e., exhibited markers of physiological stress) were more likely to die during the outbreak (DeWitte, 2015; Godde et al., 2020). The stress markers used as indicators of frailty in these studies might, in some cases, reflect exposures to malnutrition resulting from exposure to famine conditions or social inequalities in access to resources. In the post‐Black Death period, a shortage of laborers resulted in an increase in real wages and a reduction in the prices of food, goods, and housing, resulting in improved nutrition and housing conditions for individuals regardless of status (Bailey, 1996). In addition to the historical evidence, bioarchaeological investigations of trends in survivorship and of skeletal stress markers indicate an overall healthier population in London after the Black Death (DeWitte, 2014b; DeWitte, 2018).

Several studies have already investigated trends in stature in medieval and post‐medieval London. Previous research on selective mortality at the time of the Black Death has revealed that adults across all age categories were more likely to live longer after the Black Death compared to before the epidemic (DeWitte, 2014b). Additionally, DeWitte and Hughes‐Morey (2012) found that adults who were shorter in stature experienced an elevated risk of dying compared to taller individuals during the Black Death in London. DeWitte (2018) found a significant increase in tibial lengths for males in a post‐Black Death sample from London while female tibial length decreased compared to the pre‐Black Death sample. Trends in the degree of sexual stature difference before and after the Black Death, however, have not been examined for this context in London.

This study seeks to explore several research questions related to stature, SSD, and their connection to health and stress as observable through skeletal remains: What is the relationship between the environment of pre‐ and post‐Black Death London and the degree of sexual stature difference displayed in the population? Given trends in stature and SSD in this context, do the results of this study support the idea that stature can be used as a proxy for “well‐being” and health in the past and today? This study uses metric data from long bones of adult individuals from cemeteries in medieval London to compare the degree of sexual stature difference across time periods. The environment (described very generally here) is operationalized as a dichotomous variable, as poor before the Black Death and improved in the post‐Black Death period. Skeletal samples for these two time periods are comprised of individuals from London cemeteries in use prior to and after the Black Death. This study uses maximum tibial and femoral lengths as proxies for stature and sexual stature difference is defined by an index, *D*, that measures the amount of non‐overlap between distributions of a metric trait between two sexes (Bennett, 1981). In order to assess changes in stature and SSD, and their potential as indicators of well‐being, this study tests the hypothesis that due to improvements in the overall environment, the degree of sexual stature difference after the Black Death was greater than for the pre‐Black Death population in London. Previous research has indicated potential differences in frailty between in the early pre‐Black Death time period (CE 1000–1200) and late pre‐Black Death period (CE 1200–1250; DeWitte, 2015). Therefore, while SSD values in the post‐Black Death sample are expected to exceed those in both pre‐Black Death subsamples, the early pre‐Black Death values are expected to more closely resemble the post‐Black Death values owing to evidence of worsening environmental conditions in the more immediate (i.e., late) pre‐Black Death time period.

## 2 MATERIALS AND METHODS

### 2.1 Sample

This study uses data on sex, age at death, and maximum long bone length from medieval cemeteries from London, gathered from the Wellcome Osteological Research Database (WORD) at the Museum of London or collected by DeWitte. The pre‐Black Death sample includes adult individuals from St. Mary Spital Periods 14 and 15 (see details below), Guildhall Yard, and St. Nicholas Shambles, while the post‐Black Death sample is comprised of adult individuals from St. Mary Spital Period 17 and St. Mary Graces. Cemeteries were selected so that individuals within the pre‐Black Death sample experienced critical periods relevant to growth and development prior to 1348 and the majority of individuals in the post‐Black Death sample experience those critical periods after 1350. It is possible that due to the dates of first use for both St. Mary Spital (Period 17) and St. Mary Graces, a few individuals in the post‐Black Death sample experienced critical periods of growth before or during the Black Death. Given the sample size and span of cemetery use, this number is expected to be low, but nonetheless we acknowledge it as a potential source of error. For the purposes of this study, an individual is defined as an adult if they are estimated to be at least 18 years old. Adult individuals (aged >18 years old) were included in this study if they met two criteria, (1) a sex estimation of female, probable female, male or probable male, and (2) at least one tibial or femoral length measurement. Table 1 details the sample sizes of each element by London cemetery and estimated sex.

**TABLE 1:** Sample sizes by long bone, cemetery, and estimated sex

Time period	Cemetery	Tibia		Femur	
		Females	Males	Females	Males
Early pre‐BD	St. Mary Spital 14	67	101	83	116
	Guildhall Yard	8	9	4	13
	St. Nicholas Shambles	6	6	0	0
	Total	81	116	87	129
Late pre‐BD	St. Mary Spital 15	110	135	129	156
	Total	110	135	129	156
Post‐BD	St. Mary Spital 17	122	188	144	212
	St. Mary Graces	3	18	3	12
	Total	125	206	147	224

### 2.2 Pre‐Black Death sample

The hospital and priory of St. Mary Spital was established for the purpose of treating poor people, migrants to the city, and for providing a safe place for childbirth; the associated cemetery was also used for burials of members of the associated religious community (i.e., monks and lay sisters) and wealthy benefactors, and is considered to primarily be a secular cemetery (Connell et al., 2012). The St. Mary Spital cemetery is divided into four burial periods (Periods 14–17) using Bayesian radiocarbon dating and contains single internments as well as mass burials (Connell et al., 2012). Individuals included in the pre‐Black Death sample in this study are those associated with Period 14 (c.1120–1200) and Period 15 (c.1200–1250). For St. Mary Spital, type *D* burials were excluded from this study so as to not introduce error from potential plague victims. Guildhall Yard cemetery was originally the site of a lay cemetery associated with St. Lawrence Jewry dating from the 11th‐12th centuries (Bowsher et al., 2007). The northern graveyard associated with the small church of St. Nicholas Shambles also dates to the 11th‐12th centuries (Schofield, 1997). The early pre‐Black Death sample is comprised of individuals from St. Mary Spital Period 14, Guildhall Yard, and St. Nicholas Shambles while the late pre‐Black Death sample consists of St. Mary Spital Period 15 individuals.

### 2.3 Post‐Black Death sample

Individuals from St. Mary Spital Period 17 (c.1400–1539) and St. Mary Graces are included in the post‐Black Death sample. St. Mary Graces is a burial ground associated with the Cistercian abbey of St Mary Graces, in operation soon after the Black Death ended in 1350 until the Reformation in 1538 (Grainger & Phillpotts, 2011). Burials in this cemetery include lay individuals of both high and low status as well as monks.

### 2.4 Stature and demographic variables

This study used three commonly used proxies for stature, maximum tibial length, maximum femoral length, and lower limb (femur + tibia) length. Previous studies (Jantz & Jantz, 1999; Emma Pomeroy et al., 2012) have indicated that the tibia is more environmentally sensitive compared to the femur. SSD is calculated for both long bones in this study as an internal control to investigate how much SSD values fluctuate based on the long bone utilized. Estimations of stature are often achieved with regression functions using long bone lengths derived from reference samples with individuals of known stature (e.g., Robb et al., 2001; Vercellotti et al., 2011), but these estimations can be problematic. Stature estimates are complicated by variation between populations and rely upon the selection of an appropriate reference sample (Konigsberg et al., 1998). Reference samples are ideally comprised of individuals who experienced similar environmental factors (i.e., diet, genetic, and natural and built environment factors) that influence achieved adult height compared to those experienced by the population under consideration; such reference samples are rarely available for bioarchaeological samples (DeWitte & Hughes‐Morey, 2012). Though some recent research is working toward methodologies that do not rely on known group membership for stature estimation (Albanese et al., 2016), this study uses tibial and femoral lengths from WORD and collected by the second author to avoid the complications of stature estimation formulae. An osteometric board was used to measure total tibial and femoral length, with measurements recorded in millimeters. In order to maximize sample size, averages were calculated for individuals with bilateral metric data; otherwise one‐sided measurements were used.

Age at death estimates, collected from WORD, were used to select a sample comprised only of adults for this study. These ages were originally estimated by researchers at the Museum of London using four methods scoring the teeth, pelvis, and ribs (Brooks & Suchey, 1990; Brothwell, 1981; Buckberry & Chamberlain, 2002; İşcan et al., 1984, 1985; Lovejoy et al., 1985), following standards outlined by the Museum of London. Sex was estimated based on 14 total non‐metric traits of the pelvis and skull (following sexually dimorphic characteristics described by Phenice, 1969; Ferembach et al., 1980; Brothwell, 1981; Bass, 1995). Sex estimation based on the scores of pelvic and cranial traits results in five possible categories: female, probable female, intermediate, probable male, and male. For this study, individuals who were assessed as female and probable female were included in the “female” sample, and male and probable male were included in the “male” sample. Here, we would like to take the opportunity to insert a disclaimer regarding estimated sex as a variable. Many scholars have recently noted the issues with dichotomizing sex in bioarchaeological research and the ways in which a simple dichotomy ignores its continuous nature and erases intersex individuals (Astorino, 2019; DuBois & Shattuck‐Heidorn, 2021; Dunsworth, 2020; Godde et al., 2020). Estimated sex in this study, and in other bioarchaeological studies, is a variable that is subject to uncertainty. For the purposes of this study, we are dichotomizing this variable to demonstrate differences in long bone length, however, such dichotomization does not reflect the true nature of sex and may not reflect the lived experiences or expressions of those classified as estimated female or estimated male who make up the study sample.

### 2.5 Statistical analyses

Descriptive statistics of the pre‐ and post‐Black Death samples were calculated for tibial and femoral lengths for both sexes. A one‐way ANOVA analysis was performed to determine if differences in long bone lengths by estimated sex were significant across time periods. For each time period and long bone, SSD was evaluated using the Chakraborty and Majumder (1982) *D* index (based on the work of Bennett, 1981), which represents the area of non‐overlap between the male and female distributions of a metric trait. Values of the *D* index range from 0, which reflects complete overlap of the distribution and therefore no sexual difference, to 1, reflecting two distinct distributions and complete sexual difference in the phenotypic trait. Rather than just a comparison of the central tendencies, like a ratio, the *D* index allows for direct comparison between population distributions (Guatelli‐Steinberg et al., 2008). Further, the *D* index has been evaluated as a good univariate measure, and is sensitive to changes in both intersexual variances and mean differences (Marini et al., 1999). Additionally, tests of normality for each sample were conducted using the Shapiro test. All statistics were performed in R 3.6.0. *p*‐Values of .1 are considered here to indicate trends worthy of consideration.

## 3 RESULTS

Table 2 details the descriptive statistics and calculated *D* indices from tibial lengths for each time period. With the exception of the late pre‐Black Death estimated female tibial lengths (Shapiro test: *p* = .013) and the post‐Black Death estimated female tibial lengths (Shapiro test: *p* = .019), all datasets for time period and estimated sex are normally distributed. Overall, tibial lengths decreased for both estimated males and females from the early to the late pre‐BD time periods. Estimated male tibial lengths subsequently increased in the post‐Black Death time period, though for estimated females, average tibial length remained much the same. One way ANOVA analyses indicate that changes in tibial lengths for estimated males across the time periods is significant (*p* = .0542) but not for estimated females (*p* = .476).

**TABLE 2:** Descriptive statistics, *D* index (with standard deviation) and coefficient of variation for tibial lengths (in mm) by estimated sex and time period

Time period		Females	Males	*D*	SD of *D*
Early pre‐BD	Mean	335.93	364.25	0.564	0.060
	SD	18.84	17.55		
	*N*	81	116		
	V*	5.608	4.818		
Late pre‐BD	Mean	332.67	358.39	0.461	0.057
	SD	21.27	20.63		
	*N*	110	135		
	V*	6.394	5.756		
Post‐BD	Mean	332.28	362.44	0.491	0.049
	SD	20.21	20.89		
	*N*	125	206		
	V*	6.028	5.764		

In Table 3, the descriptive statistics and calculated *D* indices from femoral lengths are listed by time period. For both estimated males and estimated females, femoral length decreased from the early to the late pre‐Black Death periods and then increased again in the aftermath of the Black Death. Results of the one‐way ANOVA revealed that such changes across time are statistically significant for estimated females (*p* = .0452) as well as estimated males (*p* = .0301).

**TABLE 3:** Descriptive statistics, *D* index (with standard deviation) and coefficient of variation for femoral lengths (in mm) by estimated sex and time period

Time period		Females	Males	*D*	SD of *D*
Early pre‐BD	Mean	422.14	450.89	0.501	0.059
	SD	20.12	22.58		
	*N*	87	129		
	V*	4.766	5.008		
Late pre‐BD	Mean	416.06	448.80	0.506	0.051
	SD	23.96	23.87		
	*N*	129	156		
	V*	5.759	5.319		
Post‐BD	Mean	422.32	454.86	0.537	0.049
	SD	22.79	21.54		
	*N*	147	224		
	V*	5.396	4.736		

Calculations of the *D* index from the tibial and femoral lengths yielded somewhat disparate trends. Based on calculations from the tibia, SSD values decreased drastically from the early (*D* = 0.564) to late (*D* = 0.461) pre‐Black Death periods, and then increased again in the post‐Black Death period (*D* = 0.491). Alternatively, there is little change to the femoral *D* index values between the early pre‐Black Death period (*D* = 0.501) and the late pre‐Black Death period (*D* = 0.506). Then in the post Black Death period, we see an increase in the degree of difference for femoral lengths (*D* = 0.537).

Table 4 displays the descriptive statistics and calculated *D* indices for lower limb (femur + tibia) lengths, listed by time period. Due to small sample sizes, trends here should be interpreted with caution and ANOVA analyses were not performed to test differences of stature represented by lower limb length. However, *D* index values calculated from lower limb lengths display the same pattern as those for maximum tibial length. The degree of difference decreases from the early pre‐Black Death period (*D* = 0.878) to the late pre‐Black Death period (*D* = 0.646) and then increases in the period following the Black Death (*D* = 0.755).

**TABLE 4:** Descriptive statistics, *D* index (with standard deviation) and coefficient of variation for lower limb (femur + tibia) lengths (in mm) by estimated sex and time period

Time period		Females	Males	*D*	SD of *D*
Early pre‐BD	Mean	738.39	824.35	0.878	0.102
	SD	24.22	31.59		
	*N*	9	13		
	V*	3.28	3.83		
Late pre‐BD	Mean	745.00	824.23	0.646	0.134
	SD	37.17	49.28		
	*N*	24	13		
	V*	4.99	5.98		
Post‐BD	Mean	741.07	826.18	0.755	0.105
	SD	36.11	37.04		
	*N*	15	28		
	V*	4.87	4.48		

Coefficient of variance (V*) values are fairly consistent across time periods for the tibial and femoral lengths. Considering tibial lengths, estimated females have a larger coefficient of variance than their male counterparts for all time periods, however the value of the coefficient of variance for males fluctuates more across the three time periods. For femoral lengths, females also tend to have a larger V* value, though this pattern does not hold for the early pre‐Black Death period. The fluctuation in coefficient of variance values is also greater for estimated females across the three time periods than estimated males for the femoral length data.

## 4 DISCUSSION

This study hypothesized that both long bone length, as a proxy for stature, and degree of SSD would increase in the post‐Black Death sample due to improvements in the overall environmental and socioeconomic conditions in London after the Black Death. This hypothesis is generally supported by both the long bone length and SSD results. As expected, tibial and femoral lengths, as a proxy for height, for estimated males increased more dramatically than for estimated females in the post‐Black Death sample compared to the late pre‐Black Death sample. This finding is consistent with the idea that growth and development for males fluctuates more with environment changes compared to that of females, and that males may have benefitted more from the improved socioeconomic conditions in London after the Black Death compared to females (DeWitte, 2018). The overall degree of SSD, for both femoral and tibial data, did increase in the period following the Black Death compared to the late pre‐Black Death period, driven by more dramatic increases in male long bone lengths. However, the trends in the coefficient of variance (V*) are not consistent with this, as we overall see larger values for the females across time periods. This could be the result of a survival selection bias, wherein males who experienced growth disruption are more likely to die in childhood than their female counterparts, resulting in adult cohorts of males with less variation in stature compared to females. Additionally, due to the biological buffering mechanisms described in the introduction of this paper, even shorter females may have survived into adulthood, resulting in a larger range of coefficients of variance in females compared to males. Additionally, we cannot discount the selection bias that may be at play across time periods, as it has been documented that shorter individuals faced an increased risk of mortality during the Black Death (DeWitte & Hughes‐Morey, 2012). Historical documents suggest that economic improvements yielded a higher standard of living (Kitsikopoulos, 2002) and bioarchaeological investigations signal an increase in health and decrease in mortality risk (DeWitte, 2014a). Additionally, given the possibility that the Black Death likely had at least a short term “harvesting” effect, as increased mortality was associated with skeletal evidence of early stress events during the Black Death, this event perhaps also acted as a force of natural selection (Bos & DeWitte, 2022). Consequently, the patterns seen here from London populations before and after the Black Death, likely represent a complex interplay of improved living conditions, biological buffering, and genetic selection.

This study utilized both femoral and tibial lengths as an internal control, in addition to comparing results for total lower limb length. Data from the tibia revealed more dramatic changes in both bone length and *D* index patterns, than did the femoral data. It is interesting to note that absolute tibial long bone lengths and calculated *D* index values for the tibia displayed the same pattern across time periods. When average tibial lengths decreased for males and females, so did the *D* index value in the late pre‐Black Death period; and then in the post‐Black Death period, both average tibial lengths and the *D* index increased. For the femoral data, average male and female femoral lengths increased in the late pre‐Black Death period but the value of the *D* index rose slightly. In this instance, female femoral length decreased at a greater rate than the male femoral length. Within the framework of developmental plasticity, differential femoral and tibial growth may be explained by the distal blood flow hypothesis, originally proposed to explain the relative stunting of the tibia in utero compared to the head, trunk, and upper limbs from hypoxia induced by exposure to maternal smoking during pregnancy (Lampl et al., 2003). The circulatory system of the fetus may also privilege brain growth before leg growth since blood in the ascending aorta is more oxygen rich than that in arteries descending down the body (which also must supply blood to the placenta) so that heightened nutritional demands of the brain result in growth disruptions that increase in magnitude distally under certain conditions (Bogin & Varela‐Silva, 2010). As seen in this study, using total lower limb length can drastically decrease sample sizes relative to using maximum long bone lengths. It is therefore important to note that results here demonstrated similar stature and SSD trends for the three proxies used, with tibial and lower limb length *D* indices displaying the same pattern of change over time.

Due to the growth and development timeline of the tibia and femur, achieved adult length of these long bones can be conceptualized as an accumulation of stress exposure from in utero to late adolescence for both males and females. However, both biological (catch‐up growth) and social (migration) schemes can account for trends seen in this study regarding stature and SSD. Models of human growth that explain a reduction in growth velocity that can result in a shortened adult stature include environmental considerations like undernutrition as well as conditions like hypothyroidism or chronic inflammatory diseases (Wit & Boersma, 2002). In a metanalysis of catch up growth studies, Victora et al. (2008) found that factors like maternal height and poor maternal nutrition were most predictive of early growth failures. Catch up growth, or the improvement of growth upon later nutritional supplementation, may obscure growth affecting experiences earlier in life (Cameron et al., 2005; Clark et al., 1986) and has previously demonstrated sex specific trends (Stinson, 1985). Longitudinal studies in living populations have mostly investigated the prevalence of catch up growth during early childhood periods, (Cameron et al., 2005; Crookston et al., 2010; Desmond & Casale, 2017), finding that in some instances children stunted early in life, before 2 years of age, can catch up to their peers by age 5. However, compensatory growth may be only able to make up a portion of lost growth potential, and in multiple settings, height differences in stunted versus non‐stunted children persisted into adulthood (Victora et al., 2008). Therefore, the potential of catch‐up growth is considered limited by some researchers, since individuals tend to remain in the environment in which stunting initially occurred (Martorell et al., 1994). Since we cannot rule out the possibility of catch‐up growth, stature and consequently, sexual stature difference, may not capture the whole of environmental insults experienced during earlier growth and development in the context of pre‐ and post‐Black Death London.

Given the importance of environmental factors during the growth period, we also have to consider changes to said environment and the possibility that not all individuals in this study experienced the same urban conditions during critical periods. In addition to catch‐up growth, possible migration may also complicate the interpretation of results from this and similar studies, since the majority of growth for some individuals was likely achieved in a different living condition (rural settings) from where they were ultimately buried. Based on historical documentation, young men and women alike migrated from the surrounding rural areas to London for apprenticeships at varying ages (Klunk et al., 2019; Kowaleski, 2014). A recent genetic analysis demonstrated the mitochondrial DNA diversity in medieval London was high before, during, and after the Black Death, which could point to high relatively constant levels of female migration to the city (Klunk et al., 2019). Additionally, bioarchaeological studies using stable isotope analysis have identified potential migrations in both St. Mary Spital and St. Nicholas Shambles cemeteries (Kendall et al., 2013; Walter, 2017). Parish records indicate that in post‐Black Death London, the average age of male apprenticeship decreased from about 16 to 14 years of age, and osteological data indicates an increased presence in the city of females at younger ages than males in this time period (Lewis, 2016). If they experienced food insecurity or a significant change in living conditions, it is possible that younger female migration to London after the Black Death contributed to a less significant increase in average female adult stature relative to that of their male counterparts. In addition to rural to urban migration, immigration patterns to medieval London can be a confounding factor for this type of study. Based largely on data from the alien subsidy, a poll tax implemented for first generation non‐native born individuals moving to England, almost 7% of London's population in 1440 consisted of immigrants (Lambert, 2020). These immigrants may have contributed positively to the overall health of late medieval London with the “healthy immigrant effect” (McDonald & Kennedy, 2004), a phenomenon documented in contemporary populations wherein immigrant populations report better health than inhabitants of their sending and receiving populations. However, the research that covers such “health gaps” between immigrants and longer‐term inhabitants of receiving populations reveals that healthier immigrant populations is not ubiquitous, especially across Europe (Moullan & Jusot, 2014). Additionally, migrant or immigrant identity can yield disparate living conditions and health outcomes given possible challenges that are associated with assimilation (Bhugra, 2004).

## 5 CONCLUSION

Overall, this study demonstrates that as markers of “well‐being,” stature and sexual stature dimorphism results can be useful markers, as long as they are analyzed in the context of the environmental conditions and selection biases. Additionally, SSD indices need to be evaluated based on stature trends since previous research has revealed that height dimorphism may be an indicator of gender discrimination in regards to nutritional access and workload burdens (Moradi & Guntupalli, 2009). Therefore, an increase in SSD may indicate a relative decrease in environmental or nutritional conditions for women, rather than a relative increase in conditions for men. Indeed, stature or SSD trends alone should not be used to determine health status or changes in patterns of health overtime. Health represents a complex and multi‐dimensional process of physiological, psychological, and cultural contributions to well‐being throughout an individual's life (Frenk & Gómez‐Dantés, 2014; Molla et al., 2003). Therefore, the presence of any one marker of “well‐being” or the absence of any one marker of a disease process should not be used to determine overall health. Future bioarchaeological studies may investigate how stature and the degree of sexual stature dimorphism covaries with other markers of stress and health, such as skeletal lesions and dental carries.

## AUTHOR CONTRIBUTIONS

**Emily J. Brennan:** Data curation, Formal analysis, Methodology, Writing—Original Draft Preparation. **Sharon N. DeWitte:** Conceptualization, Methodology, Writing—Review & Editing.

## CONFLICT OF INTEREST

The authors declare no conflict of interest.

## Data Availability

Data was curated from the Wellcome Osteological Research Database (WORD) from the Museum of London Archaeological Archive, Centre for Human Bioarchaeology (https://www.museumoflondon.org.uk/collections/other‐collection‐databases‐and‐libraries/centre‐human‐bioarchaeology/osteological‐database). Curated datasets of the individuals included in the present study are available by request from the first author.
